# Supplementary material for: An intranasal live-attenuated SARS-CoV-2 vaccine limits virus transmission
Source: Nat Commun. 2024 Feb 2;15:995. doi: 10.1038/s41467-024-45348-2 (PMC10837132; doi:10.1038/s41467-024-45348-2)
Supplement: Supplementary file 1 — Supplementary Information [file 41467_2024_45348_MOESM1_ESM.pdf]

An Intranasal Live-Attenuated SARS-CoV-2 Vaccine Limits Virus Transmission

Julia M. Adler[1], Ricardo Martin Vidal[1], Christine Langner[1], Daria Vladimirova[1], Azza Abdelgawad[1], Daniela Kunecova[1], Xiaoyuan Lin[1,2], Geraldine Nouailles[3], Anne Voss[4], Sandra Kunder[4], Achim D. Gruber[4], Haibo Wu[2], Nikolaus Osterrieder[1], Dusan Kunec[1#], Jakob Trimpert[1#*]

[1]Institut für Virologie, Freie Universität Berlin, Berlin, Germany

[2]School of Life Sciences, Chongqing University, Chongqing, China

[3]Department of Infectious Diseases, Respiratory Medicine and Critical Care, Charité – Universitätsmedizin Berlin, corporate member of Freie Universität Berlin and Humboldt-Universität zu Berlin, Berlin, Germany

[4]Institut für Tierpathologie, Freie Universität Berlin, Berlin, Germany

[#]These authors jointly supervised the study

*Correspondence to: trimpert.jakob@fu-berlin.de

Table of Contents

Supplementary Fig. 1. ……………………………........ page 2-3

Supplementary Fig. 2. ……………………………........ page 4

Supplementary Fig. 3. ……………………………........ page 5

Supplementary Fig. 4. ……………………………........ page 6-7

Supplementary Fig. 5. ……………………………........ page 8-9

Supplementary Fig. 6. ……………………………........ page 10

Supplementary Fig. 7. ……………………………........ page 11

Supplementary Table 1. ..………………………………. page 12

Supplementary Table 2. ……………………………….. page 13

Supplementary Table 3. ……………………………….. page 14

Supplementary Table 4. ……………………………….. page 15

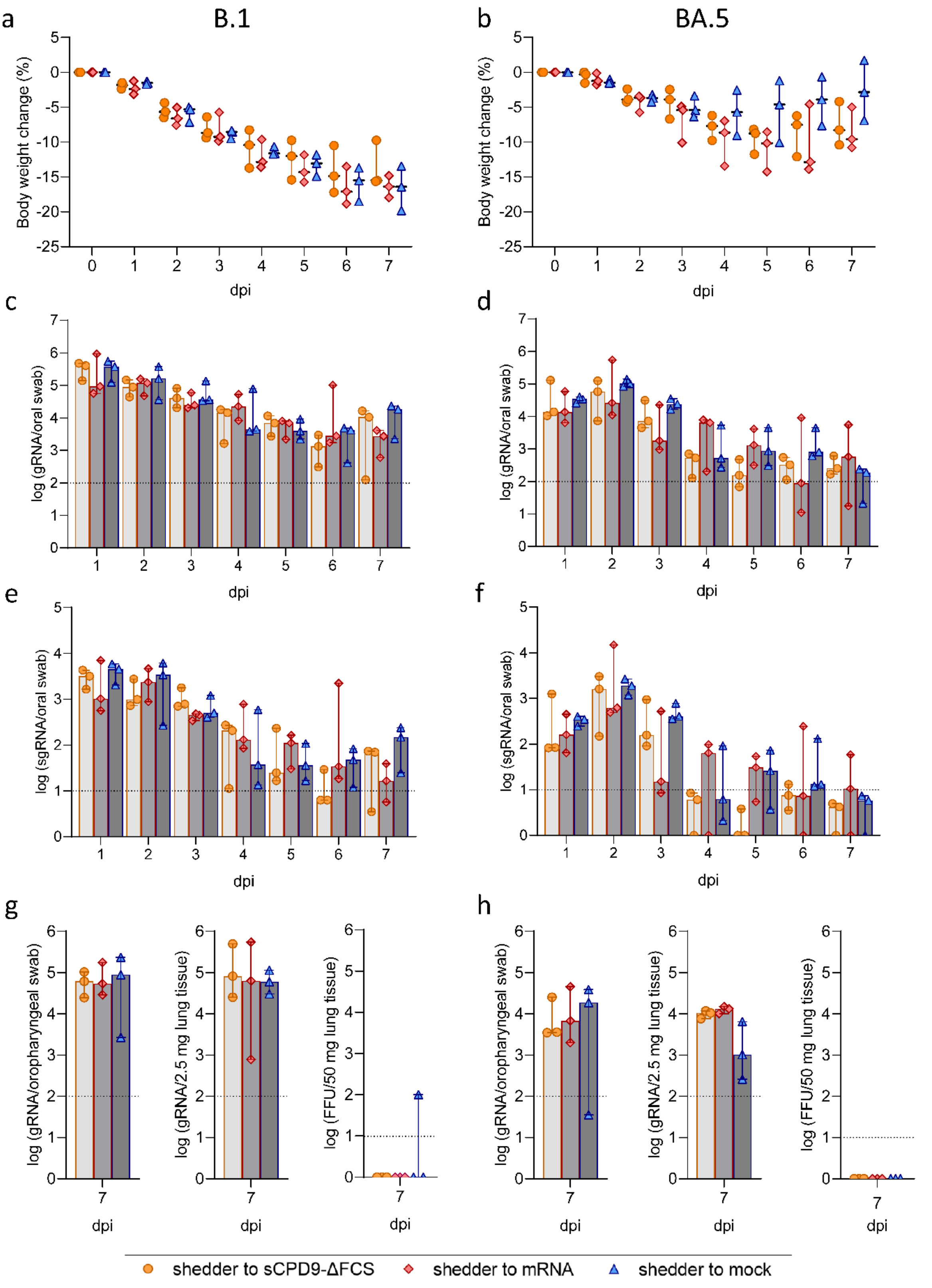

Supplementary Fig. 1. Clinical and virological outcomes in shedder animals. a, b Scatter dot plots show body weight changes in shedder hamsters (n=3) infected with SARS-CoV-2 B.1 (a) or BA.5 (b), with symbols representing individual values and thick horizontal lines showing group medians. **c, d** Viral genomic RNA (gRNA) levels in daily oral swabs of shedder hamsters infected with B.1 (c) or BA.5 (d). **e, f** Subgenomic RNA (sgRNA) copies in oral swabs of B.1- (e) and BA.5-infected (f) shedders. **g, h** gRNA levels determined 7 days post infection (dpi) in oropharyngeal swabs and

homogenized lung tissue, as well as replication-competent virus in lungs of shedder hamsters infected with B.1 (g) or BA.5 (h). (c-h) Log transformed data of shedders (n=3) are shown with individual data points represented by symbols. The bars represent the median values, and the dotted lines indicate the limits of detection. Source data are provided as a Source Data file.

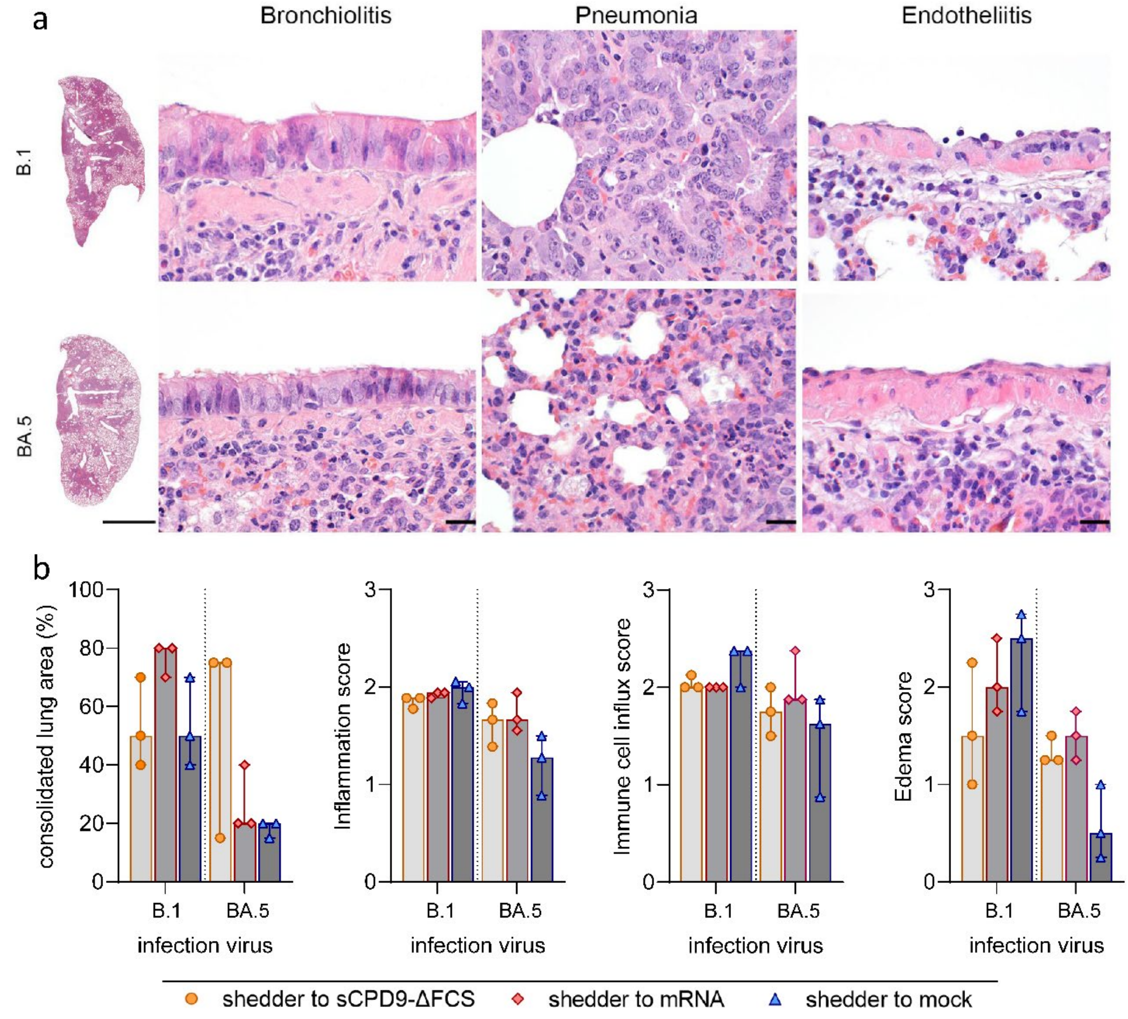

Supplementary Fig. 2. Histopathological findings in shedder hamsters infected with SARS-CoV-2 B.1 or BA.5. a Haematoxylin and eosin-stained sections of the left lung lobes obtained from shedder hamsters on day 7 after infection. Scale bars: 3 mm in the left column, and 20 µm in all other columns. **b** Semiquantitative scoring of lung consolidation is shown in percentage and all other scores are shown on a scale of 0-3. The pulmonary inflammation score includes influx of neutrophils, lymphocytes and macrophages, bronchial epithelial necrosis, bronchitis, alveolar epithelial necrosis, perivascular lymphocyte cuffs as well as pneumocyte type II hyperplasia. Immune cell influx score accounts for infiltration of lymphocytes, neutrophils and macrophages, as well as perivascular lymphocyte cuffs. Pulmonary edema score includes perivascular and alveolar edema. Symbols represent individual values (n=3) of shedder hamsters. Results are displayed as medians with range. Source data are provided as a Source Data file.

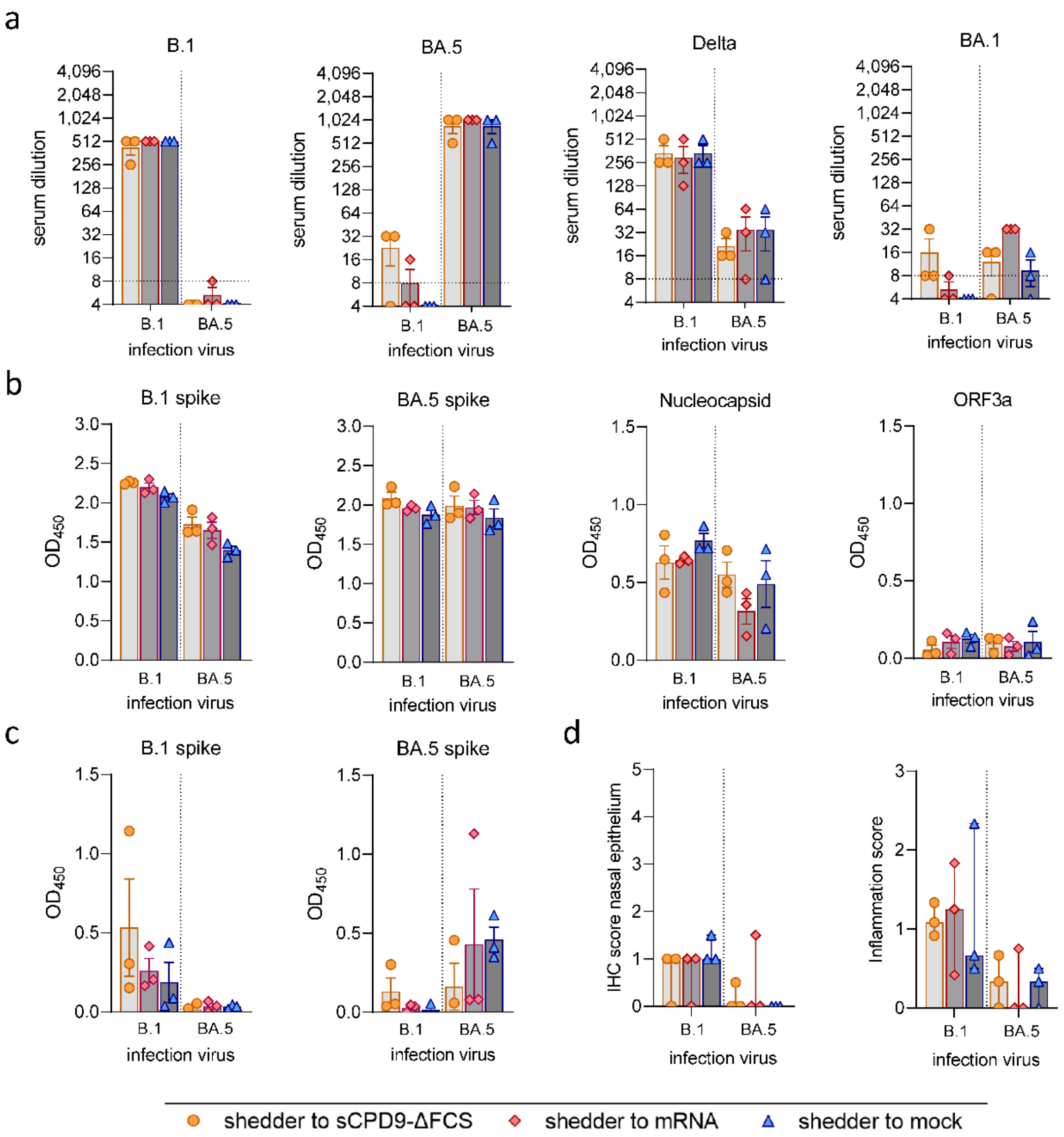

Supplementary Fig. 3. Serological findings in shedder hamsters. a Neutralizing antibody titers against SARS-CoV-2 variants B.1, BA.5, Delta and BA.1 in hamster sera collected on day 7 post infection (dpi). Individual values (n=3) are shown as symbols. The bars depict the mean, and the error bars indicate the SEM. Lower limit of detection is indicated by the dotted line (UDL=1:1,024). **b** SARS-CoV-2-specific IgG antibody levels in serum of shedder animals collected 7 dpi. Individual values of shedders (n=3) are represented by the symbols, mean ± SEM are shown for optical density (OD) measurements at 450 nm. **c** IgA antibody levels against the spike protein of SARS-CoV-2 B.1 and BA.5 in nasal washes of shedder hamsters obtained 7 dpi. Individual values (n=3), mean ± SEM are shown for OD measurements at 450 nm. **d** Quantification of SARS-CoV-2 nucleocapsid protein (N) and pathological changes in nasal epithelium of vaccinated contacts. The presence of N in nasal epithelium was detected through immunohistochemical (IHC) staining. The quantity of N and pathological changes in the epithelium were assessed using a semi-quantitative approach. Scoring of inflammatory changes considered the presence of lymphocytes and neutrophils, olfactory and respiratory epithelial necrosis, apoptosis, loss of cilia and flattened epithelial cells. Shown are the individual scores of shedders (n=3), and the median with range. Source data are provided as a Source Data file.

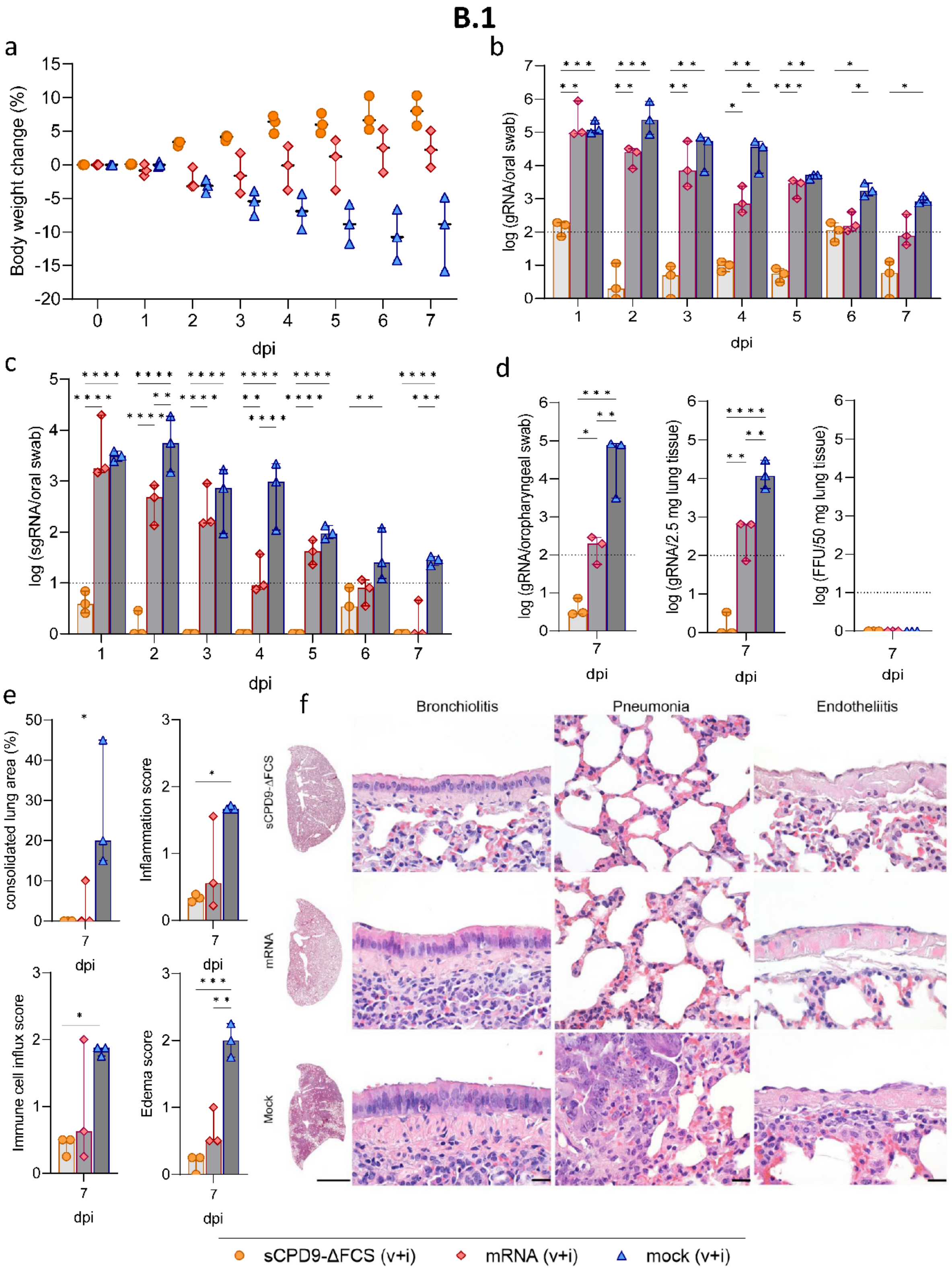

Supplementary Fig. 4. Clinical, virological and histopathological findings in vaccinated and B.1-infected animals (v+i). a Body weight change of vaccinated and B.1-infected animals (n=3). Symbols indicate individual values and thick horizontal lines showing group medians. **b, c** Viral gRNA (b) and sgRNA (c) levels in oral swabs. **d** Viral gRNA levels in oropharyngeal swabs and homogenized lung tissue, and the number of infectious virus particles present in hamster lungs at termination. **e** Histopathological scoring of inflammatory changes. The extent of consolidated lung area is shown in percentage. All other scores are shown on a scale of 0-3. Pneumonia score includes the influx of

neutrophils, lymphocytes and macrophages, bronchial epithelial necrosis, bronchitis, alveolar epithelial necrosis, perivascular lymphocyte cuffs as well as pneumocyte type II hyperplasia. Immune cell influx score accounts for the infiltration of lymphocytes, neutrophils and macrophages, as well as perivascular lymphocyte cuffs. Pulmonary edema score includes perivascular and alveolar edema. Throughout b-e, symbols represent individual values of vaccinated and B.1-infected shedders (n=3), the detection limits are indicated by dotted lines, median values are represented by bars, and the range is displayed using error bars. **b-d** Log transformed data. **b, c** Ordinary two-way ANOVA with Tukey's multiple comparisons test was performed. **d, e** Ordinary one-way ANOVA with Tukey's multiple comparisons test was conducted. * $p < 0.05$, ** $p < 0.01$, *** $p < 0.001$, and **** $p < 0.0001$. **f** Haematoxylin and eosin-stained sections of the left lung lobes of vaccinated hamsters infected with SARS-CoV-2 B.1. Scale bars: 3 mm in the left and 20 µm in all other columns. Source data are provided as a Source Data file.

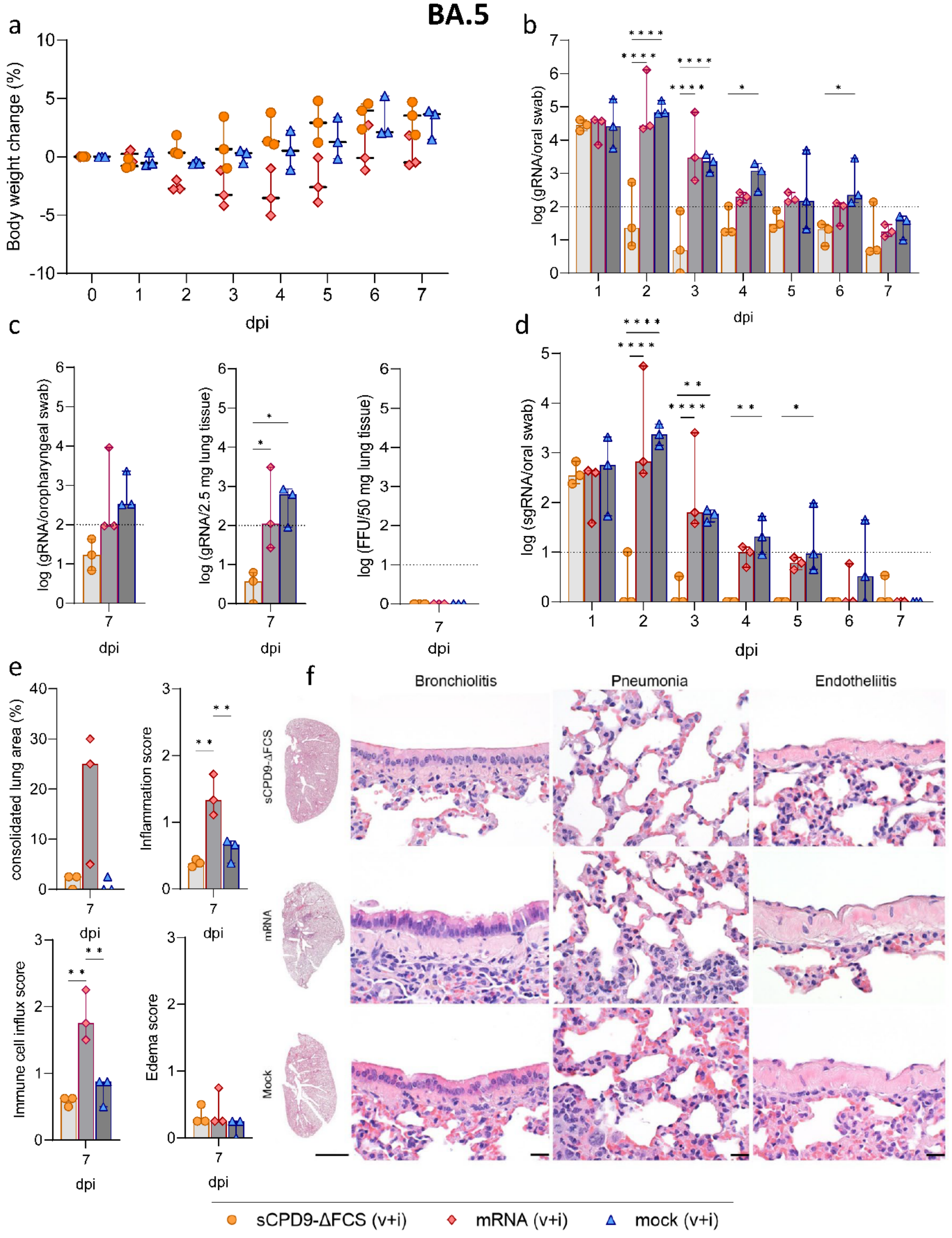

Supplementary Fig. 5. Clinical, virological and histopathological findings of vaccinated and BA.5 infected animals. a Body weight change of vaccinated and BA.5-infected animals (n=3). Symbols indicate individual values and thick horizontal lines showing group medians. **b** Viral gRNA levels in daily collected oral swabs. **c** Viral gRNA levels in oropharyngeal swabs and homogenized lung tissue, as well as the number of infectious virus particles present in hamster lungs obtained at termination, 7 days after infection (dpi). **d** sgRNA copies in oral swabs. **e** Histopathological scoring of lung sections. Lung consolidation is shown in percentage, and all other scores are shown on a scale

of 0-3, Pneumonia score includes the influx of neutrophils, lymphocytes and macrophages, bronchial epithelial necrosis, bronchitis, alveolar epithelial necrosis, perivascular lymphocyte cuffs as well as pneumocyte type II hyperplasia. Immune cell influx score accounts for infiltration of lymphocytes, neutrophils and macrophages, as well as perivascular lymphocyte cuffs. Pulmonary edema score includes perivascular and alveolar edema. Throughout b-d, log transformed data are shown, symbols represent individual values of vaccinated and BA.5-infected shedders (n=3), the detection limits are indicated by dotted lines, median values are represented by bars, and the range is displayed using error bars. **b, d** Ordinary two-way ANOVA with Tukey's multiple comparisons test was performed. **c, e** Ordinary one-way ANOVA with Tukey's multiple comparisons test was conducted. * $p < 0.05$, ** $p < 0.01$, *** $p < 0.001$, and **** $p < 0.0001$. **f** Haematoxylin and eosin-stained sections of the left lung lobes of vaccinated hamsters infected with Omicron BA.5. Scale bars: 3 mm in the left column and 20 µm in all other columns. Source data are provided as a Source Data file.

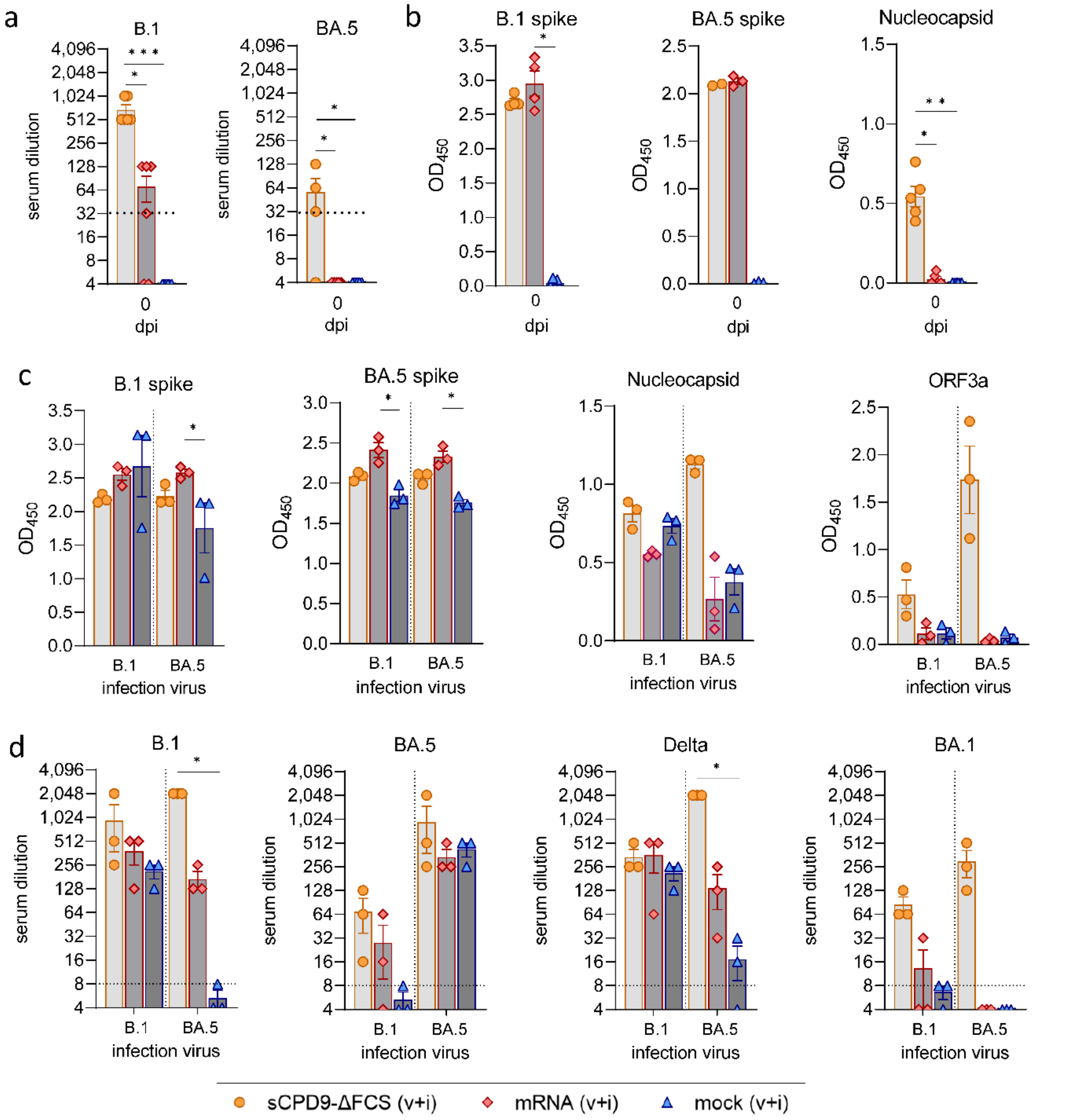

Supplementary Fig. 6. Humoral immune response in vaccinated and challenge-infected animals. a Neutralizing capacity against SARS-CoV-2 B.1 (n=6) and Omicron BA.5 (sCPD9-ΔFCS n=4, mRNA and mock n=5) in sera collected prior to infection (0 dpi). The lower limit of detection (1:32) is shown as dotted line and the upper limit is set at 1:4,096. **b, c** SARS-CoV-2-specific IgG antibody levels in sera of vaccinated and infected hamsters. The IgG levels against different SARS-CoV-2 proteins were quantified by ELISA. IgG levels against the spike protein of SARS-CoV-2 B.1 and BA.5 and the nucleocapsid protein (irrespective of the virus variant) were quantified in sera collected both before the infection (B.1 spike: sCPD9-ΔFCS n=4, mRNA n=4, mock n=5; BA.5 spike: sCPD9-ΔFCS n=2, mRNA n=3, mock n=3; Nucleocapsid: sCPD9-ΔFCS n=5, mRNA n=5, mock n=6) (b) and on day 7 after infection (n=3) (c). IgG antibody levels against the ORF3a protein were measured only in sera collected at 7 dpi, due to limited serum quantities of samples collected at 0 dpi. Values show optical density (OD) measurements obtained at 450 nm. **d** Serum neutralization titers against SARS-CoV-2 variants B.1, BA.5, Delta and BA.1 in sera collected 7 dpi (n=3). The lower limit of detection is 1:8, and the upper limit of detection is 1:1,024. In all panels (a-d) the data for vaccinated and infected animals is displayed as mean ± SEM, with individual values represented by symbols. Kruskal-Wallis test with Dunn's multiple comparisons test. * $p < 0.05$, ** $p < 0.01$, and *** $p < 0.001$. Source data are provided as a Source Data file.

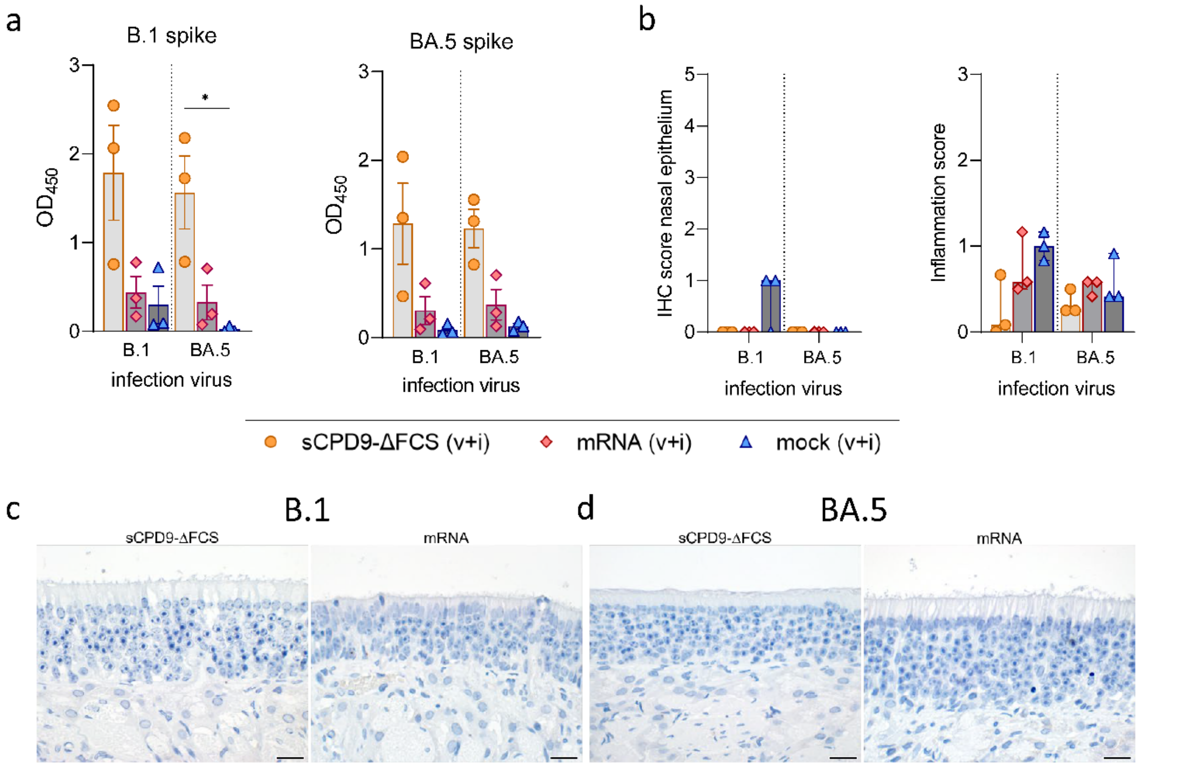

Supplementary Fig. 7. Mucosal immunity in vaccinated and challenge-infected animals. a IgA antibody levels against the spike protein of SARS-CoV-2 B.1 and BA.5 detected in nasal washes of shedder hamsters (n=3) collected 7 days after infection. The IgA levels were quantified by ELISA. The OD was measured at 450 nm. The data are displayed as mean ± SEM, with individual values represented by symbols. Kruskal-Wallis test with Dunn's multiple comparisons test. * $p < 0.05$. **b** Semi-quantitative evaluation of the presence of SARS-CoV-2 N protein in nasal epithelium of vaccinated and challenged shedders (n=3). The nucleocapsid (N) protein in nasal epithelium was detected through immunohistochemical (IHC) staining. The quantity of N protein and pathological changes in the epithelium were assessed in a semi-quantitative manner. Scoring of inflammatory changes accounts for the presence of lymphocytes, neutrophils, olfactory and respiratory epithelial necrosis, apoptosis, loss of cilia and flattened epithelial cells. Shown are the individual scores, and the median with range. **c, d** Immunohistochemical staining of SARS-CoV-2 N protein with DAB chromogen (expected brown precipitate) and hemalum counterstain (blue). Refer to Fig. 6 for the positive control. Scale bars: 20 µm. Source data are provided as a Source Data file.

	Transmission to vaccinated contacts																	
Vaccination (contacts)	sCPD9 ΔFCS																	
Infection virus (shedder)	SARS-CoV-2 B.1									Omicron BA.5								
Cage	1			2			3			4			5			6		
gRNA copies per	vaccinated contact	vaccinated contact	shedder	vaccinated contact	vaccinated contact	shedder	vaccinated contact	vaccinated contact	shedder	vaccinated contact	vaccinated contact	shedder	vaccinated contact	vaccinated contact	shedder	vaccinated contact	vaccinated contact	shedder
Oral swab 0 dpc	5.96E+01	1.00E+00	4.07E+05	1.00E+00	1.00E+00	4.76E+05	1.00E+00	1.00E+00	1.40E+05	1.00E+00	1.00E+00	1.30E+05	1.00E+00	8.30E+00	1.38E+04	2.53E+00	3.90E+00	1.06E+04
Oral swab 1 dpc	9.92E+02	3.98E+01	4.41E+04	1.33E+02	2.81E+02	1.46E+05	8.77E+02	2.71E+02	8.82E+04	3.68E+01	2.28E+02	1.24E+05	2.63E+02	1.19E+03	5.83E+04	2.85E+00	1.09E+02	7.35E+03
Oral swab 2 dpc	1.24E+02	4.98E+01	4.07E+04	8.75E+01	6.03E+01	8.08E+04	3.87E+01	3.95E+01	2.07E+04	1.36E+02	1.37E+01	4.48E+03	9.07E+00	3.53E+00	7.22E+03	1.39E+02	1.00E+00	3.16E+04
Oral swab 3 dpc	1.29E+01	3.09E+01	1.81E+04	3.18E+01	1.37E+01	1.45E+04	3.72E+01	4.59E+01	1.63E+03	6.39E+00	1.10E+01	7.08E+02	1.69E+01	1.06E+01	1.27E+02	1.00E+00	1.00E+00	5.29E+02
Oral swab 4 dpc	3.86E+01	3.73E+01	2.74E+03	2.15E+01	5.25E+01	1.16E+04	9.67E+01	6.54E+01	6.93E+03	4.78E+02	4.43E+02	6.81E+01	9.45E+00	3.71E+00	4.74E+02	1.13E+02	8.05E+01	1.56E+02
Oral swab 5 dpc	6.22E+01	4.39E+01	1.33E+03	5.30E+01	5.08E+01	2.94E+03	3.37E+01	2.91E+01	3.06E+02	3.95E+01	2.54E+01	1.13E+02	5.02E+00	1.00E+00	3.26E+02	4.89E+00	1.00E+00	5.50E+02
Oral swab 6 dpc	4.38E+00	2.90E+00	1.25E+02	1.22E+02	1.69E+01	1.65E+04	5.05E+00	3.53E+02	1.05E+04	1.03E+02	1.00E+00	6.11E+02	1.60E+02	1.00E+00	2.54E+02	1.84E+02	9.87E+01	1.68E+02
Oropharyngal swab	2.02E+02	1.26E+01	2.47E+04	1.51E+02	4.35E+01	6.14E+04	3.56E+01	2.42E+01	1.04E+05	9.66E+00	5.67E+00	2.51E+04	6.42E+00	3.11E+01	3.59E+03	3.28E+02	1.83E+02	3.47E+03
2.5mg lung tissue	3.54E+00	1.00E+00	8.24E+04	1.00E+00	2.35E+00	4.97E+05	1.00E+00	4.51E+00	2.51E+04	1.00E+00	5.13E+00	7.58E+03	2.46E-01	2.16E+00	1.18E+04	4.76E+00	1.00E+00	1.05E+04
FFU per 50 mg lung tissue	1.00E+00	1.00E+00	1.00E+00	1.00E+00	1.00E+00	1.00E+00	1.00E+00	1.00E+00	1.00E+00	1.00E+00	1.00E+00	1.00E+00	1.00E+00	1.00E+00	1.00E+00	1.00E+00	1.00E+00	1.00E+00
SNT B.1	1024	512	512	512	2048	256	256	1024	512	512	2048	4	256	512	4	256	256	4
SNT Delta	512	256	512	256	512	256	256	512	256	512	1024	16	256	512	16	256	256	32
SNT BA.1	64	8	32	32	64	8	32	64	8	8	64	4	8	32	16	32	32	16
SNT BA.5	128	8	32	32	128	32	4	128	4	32	256	512	16	128	1024	8	64	1024
Vaccination (contacts)	mRNA																	
Infection virus (shedder)	SARS-CoV-2 B.1									Omicron BA.5								
Cage	7			8			9			10			11			12		
gRNA copies per	vaccinated contact	vaccinated contact	shedder	vaccinated contact	vaccinated contact	shedder	vaccinated contact	vaccinated contact	shedder	vaccinated contact	vaccinated contact	shedder	vaccinated contact	vaccinated contact	shedder	vaccinated contact	vaccinated contact	shedder
Oral swab 0 dpc	1.00E+00	3.26E+01	9.37E+05	2.38E+01	1.00E+00	5.71E+04	1.00E+00	1.00E+00	9.32E+04	1.00E+00	1.00E+00	5.82E+04	1.00E+00	1.00E+00	6.42E+03	7.89E+00	1.00E+00	1.37E+04
Oral swab 1 dpc	1.92E+03	5.80E+02	4.81E+04	1.14E+03	2.00E+03	1.58E+05	1.66E+03	6.26E+02	1.18E+05	1.01E+03	3.73E+02	5.44E+05	1.71E+02	1.97E+02	1.12E+04	1.80E+02	6.04E+02	2.62E+04
Oral swab 2 dpc	7.96E+04	2.84E+04	2.48E+04	9.72E+03	2.25E+04	2.05E+04	5.05E+04	3.04E+04	5.85E+04	3.44E+03	2.03E+03	2.30E+04	1.46E+02	8.89E+02	9.68E+02	4.80E+01	2.02E+02	1.80E+03
Oral swab 3 dpc	2.26E+04	2.35E+03	8.29E+03	2.04E+04	2.98E+04	2.25E+04	3.08E+03	3.26E+04	5.32E+04	2.13E+04	6.88E+03	7.88E+03	1.06E+03	2.38E+03	6.51E+03	7.48E+02	3.85E+03	2.03E+02
Oral swab 4 dpc	2.01E+04	4.22E+02	8.02E+03	1.44E+04	1.76E+04	6.97E+03	9.31E+03	9.96E+03	2.19E+03	8.99E+03	7.46E+03	3.17E+02	1.58E+03	8.00E+03	1.30E+03	6.25E+03	1.97E+04	4.11E+03
Oral swab 5 dpc	1.30E+04	1.43E+03	1.74E+03	1.30E+04	1.18E+03	2.79E+03	1.28E+04	4.36E+02	1.02E+05	4.61E+03	1.15E+04	8.84E+01	1.30E+04	3.72E+03	1.11E+01	7.96E+03	2.18E+04	9.11E+03
Oral swab 6 dpc	2.51E+03	1.75E+02	6.02E+02	1.65E+04	3.26E+03	2.72E+03	2.54E+04	4.06E+03	4.10E+03	1.88E+03	1.24E+03	5.75E+02	5.48E+03	1.28E+02	1.74E+01	4.48E+02	4.12E+03	5.53E+03
Oropharyngal swab	2.30E+04	9.98E+03	5.39E+04	3.83E+05	2.14E+03	1.75E+05	6.54E+04	3.45E+04	2.90E+04	2.73E+03	8.00E+04	4.53E+04	1.13E+05	7.70E+03	2.00E+03	4.58E+04	3.72E+04	6.79E+03
2.5mg lung tissue	7.34E+03	1.83E+03	6.32E+04	5.17E+03	5.43E+01	5.51E+05	1.12E+03	1.76E+05	7.81E+02	1.43E+04	4.33E+02	1.01E+04	9.87E+00	3.69E+01	1.33E+04	1.01E+04	1.39E+04	1.50E+04
FFU per 50 mg lung tissue	1.00E+00	1.00E+00	1.00E+00	1.00E+00	1.00E+00	1.00E+00	1.00E+00	1.00E+00	1.00E+00	1.00E+00	1.00E+02	1.00E+00	1.00E+00	1.00E+00	1.00E+00	1.00E+00	5.00E+02	1.00E+00
SNT B.1	1024	2048	512	512	256	512	256	256	512	16	8	4	8	256	4	16	32	8
SNT Delta	256	256	256	512	256	128	512	256	512	16	16	8	8	128	32	16	32	64
SNT BA.1	4	4	4	4	64	4	4	32	8	4	4	32	4	8	32	4	4	32
SNT BA.5	4	8	4	4	64	4	4	32	16	128	64	1024	4	32	1024	32	16	1024
Vaccination (contacts)	Mock																	
Infection virus (shedder)	SARS-CoV-2 B.1									Omicron BA.5								
Cage	13			14			15			16			17			18		
gRNA copies per	vaccinated contact	vaccinated contact	shedder	vaccinated contact	vaccinated contact	shedder	vaccinated contact	vaccinated contact	shedder	vaccinated contact	vaccinated contact	shedder	vaccinated contact	vaccinated contact	shedder	vaccinated contact	vaccinated contact	shedder
Oral swab 0 dpc	1.16E+01	1.00E+00	5.50E+05	4.24E+00	4.61E+01	1.23E+05	4.72E+01	2.10E+00	3.77E+05	9.38E+00	1.21E+01	3.45E+04	2.69E+01	1.95E+01	4.04E+04	1.00E+00	9.20E+00	2.58E+04
Oral swab 1 dpc	4.84E+03	1.68E+03	1.63E+05	1.33E+03	3.17E+04	3.66E+04	2.28E+03	1.30E+03	3.79E+05	1.26E+01	4.30E+01	1.02E+05	3.09E+02	3.77E+01	8.45E+04	2.31E+01	4.22E+02	1.42E+05
Oral swab 2 dpc	1.27E+06	7.69E+04	1.36E+05	1.83E+05	1.95E+05	3.65E+04	1.83E+05	2.42E+05	3.42E+04	3.63E+03	4.53E+02	3.51E+04	5.62E+02	1.60E+02	2.41E+04	7.28E+02	6.21E+02	1.67E+04
Oral swab 3 dpc	6.82E+04	9.24E+04	7.95E+04	8.53E+04	9.84E+04	3.94E+03	1.92E+04	2.93E+04	4.48E+03	3.46E+04	3.17E+03	5.41E+03	6.86E+02	2.19E+04	2.70E+02	7.37E+02	2.99E+03	5.17E+02
Oral swab 4 dpc	3.02E+04	2.17E+05	2.24E+03	1.58E+04	2.19E+04	9.20E+03	6.99E+03	4.52E+04	3.99E+03	2.04E+04	1.17E+04	4.50E+03	4.98E+03	1.99E+04	8.75E+02	9.62E+03	2.02E+04	3.11E+02
Oral swab 5 dpc	1.25E+05	1.42E+04	4.88E+03	8.47E+04	1.94E+04	4.25E+03	3.59E+03	2.69E+04	4.13E+02	2.07E+03	7.04E+04	6.13E+02	2.01E+04	1.12E+04	4.52E+03	1.00E+00	1.68E+04	8.06E+02
Oral swab 6 dpc	7.96E+03	7.81E+02	2.28E+03	1.99E+04	3.47E+03	2.33E+04	1.37E+03	2.58E+05	1.84E+04	1.34E+04	1.12E+04	2.42E+02	5.84E+01	9.14E+02	2.11E+01	1.42E+04	3.21E+04	1.89E+02
Oropharyngal swab	1.80E+05	4.15E+04	2.67E+03	3.39E+04	4.77E+05	8.96E+04	8.71E+04	2.19E+04	2.36E+05	2.09E+04	7.05E+04	3.86E+04	2.72E+03	2.35E+03	3.52E+01	1.29E+05	2.79E+05	1.88E+04
2.5mg lung tissue	1.30E+06	8.27E+05	6.03E+04	1.10E+06	1.05E+06	3.04E+04	1.52E+06	3.10E+06	1.14E+05	3.63E+04	2.80E+05	2.58E+02	3.15E+03	2.48E+04	6.46E+03	1.46E+03	1.88E+02	1.03E+03
FFU per 50 mg lung tissue	2.00E+03	6.00E+03	1.00E+02	4.00E+03	2.00E+03	1.00E+00	2.00E+04	1.00E+04	1.00E+00	1.00E+02	1.00E+00	1.00E+00	1.00E+00	4.00E+03	1.00E+00	5.00E+02	1.00E+00	1.00E+00
SNT B.1	512	512	512	256	256	512	256	256	512	4	4	4	4	4	4	4	4	4
SNT Delta	256	256	256	128	256	512	256	256	256	4	32	8	4	4	64	4	4	32
SNT BA.1	4	4	4	4	4	4	4	8	4	4	4	4	4	4	8	4	4	16
SNT BA.5	4	4	4	4	4	4	4	8	4	128	256	1024	256	256	1024	128	4	512

Supplementary Table 1. Virological and serological outcomes in vaccinated contacts and their respective shedder hamsters, presented per group and cage. The table displays gRNA copy numbers detected in oral and oropharyngeal swabs, as well as in 2.5 mg of lung tissue. Oral swabs were collected daily from day 0 to day 6 post contact (dpc). Focus forming units (FFU) show the number of infectious virus particles present in 50 mg lung tissue. Negative results obtained in qPCR and plaque assays were set to 1.00E+00. The neutralizing capacity against B.1, Delta, BA.1 and BA.5 was quantified in hamster sera collected 6 dpc. Negative results obtained in serum virus neutralization assays were set to 4.

	Transmission to vaccinated contacts																	
Vaccination (contacts)	sCPD9 ΔFCS																	
Infection virus (shedder)	SARS-CoV-2 B.1									Omicron BA.5								
Cage	1			2			3			4			5			6		
sgRNA copies per	vaccinated contact	vaccinated contact	shedder	vaccinated contact	vaccinated contact	shedder	vaccinated contact	vaccinated contact	shedder	vaccinated contact	vaccinated contact	shedder	vaccinated contact	vaccinated contact	shedder	vaccinated contact	vaccinated contact	shedder
Oral swab 0 dpc	1.00E+00	1.00E+00	3.17E+03	1.00E+00	1.00E+00	4.30E+03	1.00E+00	1.00E+00	1.65E+03	1.00E+00	1.00E+00	1.25E+03	1.00E+00	1.00E+00	8.38E+01	1.00E+00	1.00E+00	8.29E+01
Oral swab 1 dpc	1.00E+00	1.00E+00	7.27E+02	1.00E+00	1.00E+00	2.74E+03	1.00E+00	1.00E+00	9.83E+02	1.00E+00	1.00E+00	3.02E+03	1.00E+00	2.28E+01	1.61E+03	1.00E+00	1.00E+00	1.49E+02
Oral swab 2 dpc	1.00E+00	2.46E+00	6.99E+02	4.27E+00	1.00E+00	1.77E+03	1.00E+00	1.00E+00	7.79E+02	2.97E+00	1.00E+00	9.14E+01	1.00E+00	1.00E+00	1.57E+02	3.57E+00	1.00E+00	9.38E+02
Oral swab 3 dpc	1.00E+00	1.00E+00	2.70E+02	1.00E+00	1.00E+00	2.07E+02	1.00E+00	1.00E+00	1.13E+01	1.00E+00	1.00E+00	6.03E+00	1.00E+00	1.00E+00	1.00E+00	1.00E+00	1.00E+00	8.42E+00
Oral swab 4 dpc	1.00E+00	1.00E+00	1.67E+01	1.00E+00	1.00E+00	2.32E+02	1.00E+00	1.00E+00	2.48E+01	1.00E+00	1.00E+00	1.00E+00	1.00E+00	1.00E+00	3.81E+00	1.00E+00	1.00E+00	1.00E+00
Oral swab 5 dpc	1.00E+00	1.00E+00	6.31E+00	1.00E+00	1.00E+00	2.92E+01	1.00E+00	1.00E+00	6.30E+00	1.00E+00	1.00E+00	3.55E+00	1.00E+00	1.00E+00	7.52E+00	1.00E+00	1.00E+00	1.31E+01
Oral swab 6 dpc	1.00E+00	1.00E+00	3.51E+00	1.00E+00	1.00E+00	7.39E+01	1.00E+00	4.54E+00	6.98E+01	1.00E+00	1.00E+00	4.96E+00	1.00E+00	1.00E+00	1.00E+00	1.00E+00	1.00E+00	4.19E+00
Mucosal anti-B.1 spike IgA (OD)	3.0378	1.6969	1.1426	1.3674	2.1981	0.3042	1.6557	1.2152	0.1518	1.6572	2.7074	0.0206	2.4497	2.2996	-0.0288	2.9540	1.7014	0.0533
Mucosal anti-BA.5 spike IgA (OD)	2.7538	1.1973	0.3013	0.8712	1.6597	0.0514	1.1561	0.8036	0.0384	1.0872	2.0997	0.0587	1.8176	1.7048	-0.0272	2.5662	1.1481	0.4557
Vaccination (contacts)	mRNA																	
Infection virus (shedder)	SARS-CoV-2 B.1									Omicron BA.5								
Cage	7			8			9			10			11			12		
sgRNA copies per	vaccinated contact	vaccinated contact	shedder	vaccinated contact	vaccinated contact	shedder	vaccinated contact	vaccinated contact	shedder	vaccinated contact	vaccinated contact	shedder	vaccinated contact	vaccinated contact	shedder	vaccinated contact	vaccinated contact	shedder
Oral swab 0 dpc	1.00E+00	1.00E+00	6.99E+03	1.00E+00	1.00E+00	5.61E+02	1.00E+00	1.00E+00	1.02E+03	1.00E+00	1.00E+00	4.47E+02	1.00E+00	1.00E+00	6.51E+01	1.00E+00	1.00E+00	1.61E+02
Oral swab 1 dpc	1.58E+01	4.54E+00	8.77E+02	1.00E+00	2.96E+01	4.62E+03	1.00E+00	4.17E+00	2.37E+03	3.30E+00	4.77E+00	1.49E+04	3.49E+00	1.00E+00	4.92E+02	1.00E+00	2.90E+00	6.20E+02
Oral swab 2 dpc	1.46E+03	5.22E+02	4.52E+02	1.41E+02	3.33E+02	3.40E+02	5.50E+02	3.84E+02	4.78E+02	8.97E+00	2.22E+01	5.21E+02	4.66E+00	1.28E+01	8.52E+00	1.00E+00	3.32E+00	1.50E+01
Oral swab 3 dpc	3.01E+02	2.50E+01	8.55E+01	2.09E+02	2.94E+02	1.30E+02	2.77E+01	6.29E+02	7.70E+02	3.62E+02	1.13E+02	9.72E+01	9.78E+00	2.67E+01	6.38E+01	1.23E+01	8.61E+01	1.00E+00
Oral swab 4 dpc	2.89E+02	6.54E+00	1.64E+02	2.92E+02	2.34E+02	1.11E+02	2.69E+02	1.59E+02	3.03E+01	1.49E+02	1.46E+02	5.46E+00	2.58E+01	9.40E+01	3.07E+01	1.32E+02	3.68E+02	5.40E+01
Oral swab 5 dpc	1.25E+02	2.01E+01	1.84E+01	3.03E+02	1.89E+01	3.40E+01	2.81E+02	1.52E+01	2.23E+03	9.49E+01	2.90E+02	7.31E+00	3.44E+02	8.75E+01	1.00E+00	2.25E+02	4.97E+02	2.44E+02
Oral swab 6 dpc	1.99E+01	1.00E+00	5.72E+00	1.47E+02	1.89E+01	1.66E+01	1.82E+02	7.76E+01	3.93E+01	1.31E+01	1.35E+01	1.05E+01	7.44E+01	4.68E+00	1.00E+00	5.16E+00	7.33E+01	5.83E+01
Mucosal anti-B.1 spike IgA (OD)	0.7420	2.6555	0.2032	0.9571	1.2974	0.1646	0.9591	2.9064	0.4144	0.1762	0.4300	0.0663	0.1000	0.4436	0.0024	0.0870	0.3024	0.0372
Mucosal anti-BA.5 spike IgA (OD)	0.5052	1.5816	0.0363	0.7562	0.8566	0.0019	0.7080	2.6350	0.0471	0.1504	0.3326	1.1299	0.0571	0.3253	0.0776	0.0633	0.2245	0.0815
Vaccination (contacts)	Mock																	
Infection virus (shedder)	SARS-CoV-2 B.1									Omicron BA.5								
Cage	13			14			15			16			17			18		
sgRNA copies per	vaccinated contact	vaccinated contact	shedder	vaccinated contact	vaccinated contact	shedder	vaccinated contact	vaccinated contact	shedder	vaccinated contact	vaccinated contact	shedder	vaccinated contact	vaccinated contact	shedder	vaccinated contact	vaccinated contact	shedder
Oral swab 0 dpc	1.00E+00	1.00E+00	5.90E+03	1.00E+00	1.00E+00	2.04E+03	1.00E+00	1.00E+00	4.56E+03	1.00E+00	1.00E+00	3.47E+02	1.00E+00	1.00E+00	4.02E+02	1.00E+00	1.00E+00	2.47E+02
Oral swab 1 dpc	7.15E+01	2.35E+01	6.16E+03	1.51E+01	2.29E+02	2.72E+02	1.36E+01	4.63E+00	3.42E+03	1.00E+00	1.00E+00	1.90E+03	5.19E+00	1.00E+00	1.18E+03	1.00E+00	8.25E+00	2.66E+03
Oral swab 2 dpc	1.24E+04	5.87E+02	1.21E+03	2.71E+03	2.02E+03	4.05E+02	1.46E+03	3.28E+03	5.03E+02	1.71E+01	9.52E+00	7.82E+02	6.49E+00	3.05E+00	3.58E+02	8.66E+00	6.23E+00	4.04E+02
Oral swab 3 dpc	6.27E+02	7.97E+02	5.87E+02	2.14E+03	1.14E+03	1.35E+01	2.43E+02	4.66E+02	3.72E+01	3.24E+02	2.42E+01	9.22E+01	3.66E+00	3.35E+02	6.20E+00	5.70E+00	1.78E+01	2.12E+00
Oral swab 4 dpc	6.55E+02	4.48E+03	1.69E+01	3.72E+02	4.67E+02	1.08E+02	1.37E+02	9.61E+02	3.60E+01	2.54E+02	1.65E+02	7.31E+01	9.46E+01	4.73E+02	2.61E+01	1.61E+02	5.72E+02	3.74E+00
Oral swab 5 dpc	2.16E+03	2.60E+02	8.31E+01	1.20E+03	2.09E+02	4.80E+01	7.34E+01	6.45E+02	1.20E+01	5.85E+01	1.42E+03	1.20E+01	3.16E+02	2.83E+02	1.32E+02	1.00E+00	3.87E+02	1.32E+01
Oral swab 6 dpc	7.07E+01	1.08E+01	2.49E+01	5.59E+02	1.61E+01	2.40E+02	1.48E+01	1.85E+03	1.49E+02	1.40E+02	1.26E+02	7.34E+00	1.00E+00	1.44E+01	1.00E+00	1.39E+02	5.30E+02	5.82E+00
Mucosal anti-B.1 spike IgA (OD)	0.0124	0.2662	0.4383	0.1035	-0.0047	0.0369	-0.0043	0.6170	0.0862	-0.0274	-0.0318	0.0450	0.0417	-0.0078	0.0339	-0.0310	-0.0163	0.0169
Mucosal anti-BA.5 spike IgA (OD)	-0.0090	0.0352	0.0541	0.0210	-0.0279	-0.0193	-0.0272	0.0627	-0.0133	-0.0215	-0.0181	0.6148	0.1062	0.1219	0.4124	-0.0093	-0.0093	0.3491

Supplementary Table 2. Virological results and mucosal immunity of vaccinated contacts and infected shedders displayed per group and cage. The table shows sgRNA copies detected in oral swabs and SARS-specific IgA levels against B.1 and BA.5 spike protein in nasal washes obtained at 6 days post contact (dpc), measured as optical density (OD) at 450 nm. Negative results obtained in qPCR assays were set to 1.00E+00.

	Transmission to naive contacts																	
Vaccination (shedder)	sCPD9 ΔFCS																	
Infection virus (shedder)	SARS-CoV-2 B.1									Omicron BA.5								
Cage	1			2			3			4			5			6		
gRNA copies per	naive contact	naive contact	vaccinated + infected	naive contact	naive contact	vaccinated + infected	naive contact	naive contact	vaccinated + infected	naive contact	naive contact	vaccinated + infected	naive contact	naive contact	vaccinated + infected	naive contact	naive contact	vaccinated + infected
Oral swab 0 dpc	1.00E+00	1.00E+00	1.93E+02	3.67E+00	5.78E+00	7.29E+01	1.47E+00	1.00E+00	1.64E+02	2.78E+00	4.28E+00	4.03E+04	1.77E+00	2.45E+00	2.82E+04	1.86E+00	4.33E+00	1.89E+04
Oral swab 1 dpc	1.00E+00	2.71E+00	1.99E+00	1.00E+00	1.00E+00	1.14E+01	1.39E+00	1.00E+00	1.00E+00	1.00E+00	1.00E+00	2.27E+01	1.00E+00	1.00E+00	6.59E+00	3.45E+00	2.36E+00	5.36E+02
Oral swab 2 dpc	4.32E+00	7.68E+00	9.22E+00	1.82E+00	1.00E+00	5.02E+00	1.00E+00	1.00E+00	1.00E+00	3.86E+01	6.14E+01	4.90E+00	1.00E+00	1.00E+00	1.01E+00	1.00E+00	3.42E+03	7.33E+01
Oral swab 3 dpc	1.06E+01	4.36E+01	6.42E+00	1.18E+01	7.64E+00	1.26E+01	5.22E+01	3.28E+01	1.03E+01	4.42E+00	4.39E+00	1.73E+01	7.92E+01	3.73E+01	1.03E+02	2.84E+01	3.18E+03	1.71E+01
Oral swab 4 dpc	9.11E+00	7.43E+00	5.63E+00	6.93E+00	4.97E+00	3.11E+00	1.00E+00	7.19E+00	8.01E+00	1.61E+00	2.67E+01	3.02E+01	1.00E+00	1.67E+00	2.18E+01	2.65E+00	2.69E+03	7.57E+01
Oral swab 5 dpc	2.04E+01	5.64E+01	1.90E+02	4.11E+01	8.64E+00	5.05E+01	1.09E+01	6.63E+01	1.14E+02	2.15E+01	1.27E+01	2.93E+01	5.01E+00	1.24E+01	6.40E+00	8.34E+00	4.51E+04	2.07E+01
Oral swab 6 dpc	6.24E+00	3.61E+00	1.00E+00	1.00E+00	2.56E+00	1.29E+01	8.20E+00	2.80E+00	5.84E+00	3.22E+00	4.09E+00	4.90E+00	3.93E+00	3.90E+00	4.51E+00	2.68E+02	9.55E+04	1.36E+02
Oropharyngal swab	9.47E+00	1.36E+00	3.02E+00	1.42E+00	3.09E+00	2.79E+00	4.73E+00	3.76E+00	7.27E+00	1.92E+00	5.98E+00	1.70E+01	4.94E+00	2.53E+00	6.86E+00	8.45E+04	3.75E+05	4.31E+01
2.5mg lung tissue	1.00E+00	1.00E+00	3.40E+00	1.00E+00	1.00E+00	1.00E+00	1.00E+00	1.00E+00	1.00E+00	1.00E+00	1.00E+00	1.00E+00	1.00E+00	1.00E+00	6.26E+00	2.07E+02	8.03E+05	3.75E+00
FFU per 50 mg lung tissue	1.00E+00	1.00E+00	1.00E+00	1.00E+00	1.00E+00	1.00E+00	1.00E+00	1.00E+00	1.00E+00	1.00E+00	1.00E+00	1.00E+00	1.00E+00	1.00E+00	1.00E+00	1.00E+00	8.00E+04	1.00E+00
SNT B.1 6 dpc	4	4	256	4	4	512	4	4	2048	4	4	2048	4	4	2048	4	4	2048
SNT B.1 0 dpc			512			512			1024			512			1024			512
SNT Delta	4	4	256	4	4	256	4	4	512	4	4	2048	4	4	2048	4	4	2048
SNT BA.1	4	4	64	4	4	128	4	4	64	4	4	128	4	4	512	4	4	256
SNT BA.5 6 dpc	4	4	16	4	4	64	4	4	128	4	4	512	4	4	2048	4	16	256
SNT BA.5 0 dpc			32			64			n.t.			128			n.t.			4
Vaccination (shedder)	mRNA																	
Infection virus (shedder)	SARS-CoV-2 B.1									Omicron BA.5								
Cage	7			8			9			10			11			12		
gRNA copies per	naive contact	naive contact	vaccinated + infected	naive contact	naive contact	vaccinated + infected	naive contact	naive contact	vaccinated + infected	naive contact	naive contact	vaccinated + infected	naive contact	naive contact	vaccinated + infected	naive contact	naive contact	vaccinated + infected
Oral swab 0 dpc	1.00E+00	1.00E+00	9.94E+04	4.13E+00	4.75E+00	8.85E+05	1.00E+00	1.00E+00	9.13E+04	1.00E+00	1.00E+00	4.04E+04	1.00E+00	1.00E+00	7.16E+03	1.13E+00	2.75E+00	3.72E+04
Oral swab 1 dpc	2.76E+01	8.12E+01	2.55E+04	6.35E+03	1.02E+02	8.13E+03	1.92E+02	1.52E+02	3.21E+04	7.85E+01	1.15E+01	2.67E+04	5.55E+00	1.18E+01	1.29E+06	6.69E+01	6.44E+02	2.22E+04
Oral swab 2 dpc	5.50E+01	2.51E+02	5.45E+04	1.04E+05	3.87E+05	7.11E+03	3.09E+02	9.74E+04	2.37E+03	1.79E+03	2.54E+02	6.13E+02	4.21E+02	2.09E+01	6.80E+04	1.20E+04	4.30E+00	2.95E+03
Oral swab 3 dpc	1.94E+02	9.17E+04	2.39E+03	6.86E+04	1.00E+05	7.12E+02	1.29E+02	2.38E+05	3.94E+02	2.07E+04	3.01E+03	1.28E+02	1.94E+04	8.43E+02	1.94E+02	1.23E+04	1.21E+04	2.66E+02
Oral swab 4 dpc	2.77E+05	1.79E+05	3.54E+03	1.11E+04	9.68E+03	3.00E+03	3.51E+05	4.01E+04	1.01E+03	1.31E+04	1.30E+04	1.39E+02	2.62E+04	8.07E+02	2.64E+02	2.27E+03	8.03E+04	1.58E+02
Oral swab 5 dpc	2.97E+04	1.84E+04	1.09E+02	2.13E+04	1.26E+03	1.55E+02	2.88E+04	2.09E+04	4.04E+02	9.02E+03	1.43E+04	2.63E+01	5.60E+03	2.55E+03	1.28E+02	3.01E+03	1.70E+04	1.04E+02
Oral swab 6 dpc	7.80E+03	2.50E+04	3.41E+02	6.60E+02	9.58E+03	7.67E+01	3.69E+04	1.41E+04	4.16E+01	1.16E+03	1.46E+04	1.75E+01	3.76E+01	8.98E+03	1.26E+01	4.93E+01	2.28E+03	2.86E+01
Oropharyngal swab	4.98E+05	8.07E+05	5.66E+01	4.64E+04	1.48E+05	1.97E+02	3.00E+05	6.76E+05	2.89E+02	6.29E+04	2.36E+05	9.12E+03	4.00E+04	7.72E+03	9.40E+01	5.40E+04	8.68E+04	9.26E+01
2.5mg lung tissue	1.18E+06	1.03E+07	7.24E+01	6.87E+05	8.60E+05	6.43E+02	5.08E+06	2.24E+07	6.57E+02	4.02E+03	5.74E+02	1.10E+02	2.69E+04	3.07E+02	3.06E+03	2.00E+04	3.69E+04	2.70E+01
FFU per 50 mg lung tissue	3.00E+03	6.00E+04	1.00E+00	1.00E+03	5.00E+02	1.00E+00	2.00E+05	1.00E+05	1.00E+00	1.00E+00	1.00E+00	1.00E+00	1.00E+02	2.50E+02	1.00E+00	1.00E+02	1.30E+03	1.00E+00
SNT B.1 6 dpc	32	16	512	512	256	128	4	64	512	4	4	256	4	4	128	4	4	128
SNT B.1 0 dpc			128			4			128			128			32			4
SNT Delta	64	8	512	512	256	64	4	128	512	4	4	256	4	4	128	4	4	32
SNT BA.1	4	4	32	32	4	4	4	4	4	4	4	4	4	4	4	4	4	4
SNT BA.5 6 dpc	4	4	64	4	4	4	4	4	16	256	128	256	64	4	512	256	4	256
SNT BA.5 0 dpc			n.t.			4			4			4			4			4
Vaccination (shedder)	Mock																	
Infection virus (shedder)	SARS-CoV-2 B.1									Omicron BA.5								
Cage	13			14			15			16			17			18		
gRNA copies per	naive contact	naive contact	vaccinated + infected	naive contact	naive contact	vaccinated + infected	naive contact	naive contact	vaccinated + infected	naive contact	naive contact	vaccinated + infected	naive contact	naive contact	vaccinated + infected	naive contact	naive contact	vaccinated + infected
Oral swab 0 dpc	1.00E+00	1.00E+00	9.72E+04	1.09E+00	3.70E+01	2.29E+05	1.00E+00	1.00E+00	1.18E+05	1.71E+00	1.00E+00	2.58E+04	1.00E+00	1.00E+00	5.65E+03	1.00E+00	2.86E+00	1.72E+05
Oral swab 1 dpc	1.49E+04	4.40E+03	8.48E+05	7.09E+03	1.77E+04	2.38E+05	3.88E+03	9.64E+03	8.78E+04	1.11E+02	3.87E+01	6.65E+04	9.67E+01	4.21E+01	6.31E+04	1.27E+02	2.15E+01	1.57E+05
Oral swab 2 dpc	2.37E+05	3.12E+05	7.21E+04	1.15E+05	1.82E+05	6.64E+03	1.97E+05	4.59E+05	5.50E+04	5.44E+04	3.27E+02	1.08E+03	4.24E+04	2.27E+03	3.72E+03	3.68E+04	3.16E+04	2.28E+03
Oral swab 3 dpc	6.91E+04	5.04E+05	5.93E+03	7.03E+04	1.91E+05	5.29E+04	1.41E+05	2.76E+05	3.68E+04	8.28E+04	2.71E+03	1.96E+03	2.31E+04	1.66E+04	1.19E+03	5.69E+03	2.32E+04	2.88E+02
Oral swab 4 dpc	7.76E+03	2.16E+04	5.20E+03	1.16E+04	2.60E+04	5.34E+03	2.64E+03	7.90E+04	3.95E+03	3.75E+04	2.05E+03	2.15E+01	1.94E+04	1.07E+05	1.46E+02	2.39E+04	1.50E+04	4.97E+03
Oral swab 5 dpc	3.79E+04	8.70E+03	1.23E+03	2.71E+04	2.03E+04	1.75E+03	5.84E+03	1.29E+04	3.00E+03	2.62E+04	3.08E+03	2.85E+03	5.73E+04	1.04E+04	2.22E+02	8.41E+03	6.25E+03	1.32E+02
Oral swab 6 dpc	5.73E+03	9.33E+03	8.34E+02	1.47E+04	5.60E+02	1.21E+03	1.83E+03	1.68E+03	7.54E+02	1.74E+03	3.34E+03	3.82E+01	6.21E+02	1.43E+03	9.86E+00	1.69E+03	3.56E+02	5.17E+01
Oropharyngal swab	3.52E+04	2.29E+05	3.13E+03	3.59E+05	1.81E+05	7.84E+04	5.99E+04	8.78E+05	8.35E+04	2.65E+04	1.15E+05	2.32E+03	2.42E+04	3.81E+04	3.33E+02	3.48E+04	1.39E+04	3.27E+02
2.5mg lung tissue	4.57E+04	2.34E+05	1.17E+04	1.80E+05	9.30E+05	2.95E+04	3.74E+05	1.25E+06	5.43E+03	9.62E+04	1.35E+03	8.57E+02	2.24E+04	5.32E+03	6.22E+02	1.55E+05	2.49E+02	9.07E+01
FFU per 50 mg lung tissue	1.00E+02	6.00E+02	1.00E+00	1.00E+02	1.00E+03	1.00E+00	2.00E+02	5.00E+02	1.00E+00	4.00E+02	1.00E+00	1.00E+00	1.00E+00	1.00E+03	1.00E+00	2.00E+03	1.00E+00	1.00E+00
SNT B.1 6 dpc	256	256	256	256	256	128	256	256	256	4	4	4	4	4	4	4	4	8
SNT B.1 0 dpc			4			4			4			4			4			4
SNT Delta	256	256	256	512	256	256	256	512	128	4	4	4	4	4	16	4	4	32
SNT BA.1	4	4	8	4	16	4	4	32	8	4	4	4	4	4	4	4	4	4
SNT BA.5 6 dpc	4	4	4	4	4	4	4	8	8	512	4	512	512	256	256	256	256	512
SNT BA.5 0 dpc			n.t.			4			4			4			4			4

Supplementary Table 3. Virological and serological outcomes of naïve contacts and their respective vaccinated and infected shedder hamsters, shown per group and cage. The table displays gRNA copy numbers detected in oral and oropharyngeal swabs, as well as in 2.5 mg lung tissue. Oral swabs were collected daily from day 0 to day 6 post contact (dpc). Focus forming units (FFU) show the number of infectious virus particles present in 50 mg of lung tissue, determined by plaque assay. Negative results obtained in qPCR and plaque assays were set to 1.00E+00. The neutralizing capacity against B.1, Delta, BA.1 and BA.5 was quantified in hamster sera collected 0 and 6 dpc. Negative results obtained in serum virus neutralization assays were set to 4. Samples labelled as "n.t." were not tested due to limited serum quantities.

	Transmission to naive contacts																	
Vaccination (shedder)	sCPD9 ΔFCS																	
Infection virus (shedder)	SARS-CoV-2 B.1									Omicron BA.5								
Cage	1			2			3			4			5			6		
sgRNA copies per	naive contact	naive contact	vaccinated + infected	naive contact	naive contact	vaccinated + infected	naive contact	naive contact	vaccinated + infected	naive contact	naive contact	vaccinated + infected	naive contact	naive contact	vaccinated + infected	naive contact	naive contact	vaccinated + infected
Oral swab 0 dpc	1.00E+00	1.00E+00	6.87E+00	1.00E+00	1.00E+00	2.56E+00	1.00E+00	1.00E+00	3.88E+00	1.00E+00	1.00E+00	6.69E+02	1.00E+00	1.00E+00	3.51E+02	1.00E+00	1.00E+00	2.40E+02
Oral swab 1 dpc	1.00E+00	1.00E+00	1.00E+00	1.00E+00	1.00E+00	2.87E+00	1.00E+00	1.00E+00	1.00E+00	1.00E+00	1.00E+00	1.00E+00	1.00E+00	1.00E+00	1.00E+00	1.00E+00	1.00E+00	1.01E+01
Oral swab 2 dpc	1.00E+00	1.00E+00	1.00E+00	1.00E+00	1.00E+00	1.00E+00	1.00E+00	1.00E+00	1.00E+00	1.00E+00	4.79E+00	1.00E+00	1.00E+00	1.00E+00	1.00E+00	1.00E+00	3.58E+01	3.27E+00
Oral swab 3 dpc	1.00E+00	1.00E+00	1.00E+00	1.00E+00	1.00E+00	1.00E+00	1.00E+00	1.00E+00	1.00E+00	2.57E+00	1.00E+00	1.00E+00	1.00E+00	1.00E+00	1.00E+00	1.00E+00	5.55E+01	1.00E+00
Oral swab 4 dpc	1.00E+00	1.00E+00	1.00E+00	1.00E+00	1.00E+00	1.00E+00	1.00E+00	1.00E+00	1.00E+00	1.00E+00	1.00E+00	1.00E+00	1.00E+00	1.00E+00	1.00E+00	1.00E+00	2.82E+01	1.00E+00
Oral swab 5 dpc	1.00E+00	1.00E+00	8.13E+00	1.00E+00	1.00E+00	1.00E+00	1.00E+00	3.20E+00	3.45E+00	1.00E+00	1.00E+00	1.00E+00	1.00E+00	1.00E+00	1.00E+00	1.00E+00	1.03E+03	1.00E+00
Oral swab 6 dpc	1.00E+00	1.00E+00	1.00E+00	1.00E+00	1.00E+00	1.00E+00	1.00E+00	1.00E+00	1.00E+00	1.00E+00	1.00E+00	1.00E+00	1.00E+00	1.00E+00	1.00E+00	1.00E+00	2.21E+03	3.40E+00
Mucosal anti-B.1 spike IgA (OD)	-0.0313	-0.0306	2.5460	-0.0292	-0.0294	2.0666	-0.0107	-0.0285	0.7557	-0.0301	-0.0151	0.7846	-0.0306	-0.0278	2.1817	-0.0296	-0.0304	1.7255
Mucosal anti-BA.5 spike IgA (OD)	-0.0296	-0.0272	2.0410	-0.0267	-0.0282	1.3512	-0.0090	-0.0273	0.4651	-0.0298	-0.0123	0.8246	-0.0272	-0.0252	1.5550	-0.0288	-0.0266	1.3133
Vaccination (shedder)	mRNA																	
Infection virus (shedder)	SARS-CoV-2 B.1									Omicron BA.5								
Cage	7			8			9			10			11			12		
sgRNA copies per	naive contact	naive contact	vaccinated + infected	naive contact	naive contact	vaccinated + infected	naive contact	naive contact	vaccinated + infected	naive contact	naive contact	vaccinated + infected	naive contact	naive contact	vaccinated + infected	naive contact	naive contact	vaccinated + infected
Oral swab 0 dpc	1.00E+00	1.00E+00	1.79E+03	1.00E+00	1.00E+00	1.97E+04	1.00E+00	1.00E+00	1.48E+03	1.00E+00	1.00E+00	4.00E+02	1.00E+00	1.00E+00	3.86E+01	1.00E+00	1.00E+00	4.35E+02
Oral swab 1 dpc	5.66E+00	1.00E+00	4.83E+02	5.08E+01	1.00E+00	1.36E+02	3.68E+00	5.48E+00	8.23E+02	2.31E+00	1.00E+00	6.61E+02	1.00E+00	1.00E+00	5.53E+04	1.00E+00	2.26E+01	3.87E+02
Oral swab 2 dpc	6.30E-01	6.80E+00	8.98E+02	2.12E+03	4.41E+03	1.50E+02	6.55E+00	1.29E+03	1.59E+02	4.79E+01	6.52E+00	3.83E+01	1.87E+01	7.78E+00	2.54E+03	1.83E+02	1.00E+00	6.29E+01
Oral swab 3 dpc	1.00E+00	1.30E+03	3.67E+01	9.95E+02	1.15E+03	9.04E+00	3.85E+00	3.57E+03	7.47E+00	5.33E+02	1.01E+02	4.99E+00	1.95E+02	4.08E+01	1.28E+01	3.19E+02	1.91E+02	1.01E+01
Oral swab 4 dpc	9.39E+03	4.55E+03	6.92E+01	2.17E+02	1.58E+02	4.15E+01	9.07E+03	1.28E+03	2.31E+01	7.93E+02	3.24E+02	4.43E+00	2.52E+03	3.63E+02	7.79E+00	1.31E+02	2.26E+03	6.10E+00
Oral swab 5 dpc	1.00E+03	2.01E+02	8.05E+00	4.94E+02	1.79E+01	3.53E+00	7.15E+02	5.46E+02	1.14E+01	2.75E+02	3.50E+02	1.00E+00	2.53E+02	7.12E+02	5.85E+00	1.55E+02	5.96E+02	1.00E+00
Oral swab 6 dpc	1.54E+02	3.90E+02	4.54E+00	1.01E+01	1.44E+02	1.00E+00	7.01E+02	2.55E+02	1.00E+00	3.26E+01	2.30E+02	1.00E+00	1.00E+00	2.59E+03	1.00E+00	1.00E+00	5.56E+01	1.00E+00
Mucosal anti-B.1 spike IgA (OD)	-0.0012	0.0067	0.1689	0.0213	0.0814	0.7770	0.0040	0.0463	0.3734	0.0104	0.0033	0.0761	0.0068	0.0188	0.1946	0.0108	0.0070	0.7082
Mucosal anti-BA.5 spike IgA (OD)	-0.0192	-0.0187	0.0941	-0.0196	-0.0173	0.6101	-0.0174	0.0014	0.2125	0.2125	0.0511	0.1276	0.0165	0.0016	0.2810	0.0212	-0.0106	0.7049
Vaccination (shedder)	Mock																	
Infection virus (shedder)	SARS-CoV-2 B.1									Omicron BA.5								
Cage	13			14			15			16			17			18		
sgRNA copies per	naive contact	naive contact	vaccinated + infected	naive contact	naive contact	vaccinated + infected	naive contact	naive contact	vaccinated + infected	naive contact	naive contact	vaccinated + infected	naive contact	naive contact	vaccinated + infected	naive contact	naive contact	vaccinated + infected
Oral swab 0 dpc	1.00E+00	1.00E+00	3.12E+03	1.00E+00	1.00E+00	3.89E+03	1.00E+00	1.00E+00	2.45E+03	1.00E+00	1.00E+00	5.72E+02	1.00E+00	1.00E+00	5.36E+01	1.00E+00	1.00E+00	2.09E+03
Oral swab 1 dpc	2.67E+02	8.73E+01	1.88E+04	5.49E+01	2.48E+02	5.63E+03	7.09E+01	1.77E+02	1.50E+03	3.90E+00	3.32E+00	2.36E+03	4.02E+00	1.00E+00	1.42E+03	2.62E+00	1.00E+00	3.79E+03
Oral swab 2 dpc	4.25E+03	6.24E+03	1.69E+03	1.60E+03	3.21E+03	9.32E+01	4.02E+03	8.97E+03	7.28E+02	6.91E+02	5.49E+00	4.07E+01	7.32E+02	3.33E+01	7.12E+01	3.27E+02	4.68E+02	5.86E+01
Oral swab 3 dpc	1.95E+03	1.43E+04	1.09E+02	1.98E+03	7.94E+03	2.18E+03	4.45E+03	8.56E+03	9.70E+02	4.39E+03	1.37E+02	5.20E+01	7.33E+02	6.58E+02	2.07E+01	1.27E+02	9.86E+02	9.09E+00
Oral swab 4 dpc	1.33E+02	8.24E+02	9.45E+01	3.89E+02	7.70E+02	1.35E+02	7.88E+01	1.46E+03	7.42E+01	1.14E+03	8.73E+01	4.48E+00	7.58E+02	3.26E+03	9.41E+00	8.45E+02	9.58E+02	9.64E+01
Oral swab 5 dpc	5.16E+02	1.84E+02	1.23E+01	3.36E+02	2.25E+02	2.53E+01	3.69E+01	1.01E+02	1.20E+02	5.10E+02	8.97E+01	4.51E+01	1.69E+03	2.01E+02	3.29E+00	1.22E+02	1.10E+02	1.00E+00
Oral swab 6 dpc	8.02E+01	1.61E+02	2.20E+01	2.63E+02	2.62E+01	2.91E+01	4.35E+01	7.98E+01	3.32E+01	3.36E+01	8.97E+01	1.00E+00	1.63E+01	3.24E+01	1.00E+00	7.83E+01	1.31E+01	1.00E+00
Mucosal anti-B.1 spike IgA (OD)	0.0723	0.0235	0.7220	0.1600	0.0922	0.0938	0.0321	0.0308	0.0772	-0.0022	-0.0048	0.0062	0.0207	0.0226	0.0582	0.0471	0.0110	0.0016
Mucosal anti-BA.5 spike IgA (OD)	0.0198	0.0092	0.1550	0.0774	0.0461	0.0637	0.0418	0.0465	0.0417	0.0731	0.0451	0.1801	0.0518	0.0318	0.1280	0.0588	0.0114	0.0755

Supplementary Table 4. Virological results and mucosal immunity of naïve contacts and their respective vaccinated and infected shedder animals, presented per group and cage. The table shows sgRNA copies detected in oral swabs, and SARS-CoV-2-specific IgA levels against B.1 and BA.5 spike protein in nasal washes obtained at 6 days post contact (dpc), measured in optical density (OD) at 450 nm. Negative results obtained in qPCR assays were set to 1.00E+00.
