# Supplementary material for: An intranasal live-attenuated SARS-CoV-2 vaccine limits virus transmission
Source: Nat Commun. 2024 Feb 2;15:995. doi: 10.1038/s41467-024-45348-2 (PMC10837132; doi:10.1038/s41467-024-45348-2)
Supplement: Supplementary file 3 — Reporting Summary [file 41467_2024_45348_MOESM3_ESM.pdf]

Corresponding author(s): Jakob Trimpert

Last updated by author(s): July 26, 2023

Reporting Summary

Nature Portfolio wishes to improve the reproducibility of the work that we publish. This form provides structure for consistency and transparency in reporting. For further information on Nature Portfolio policies, see our Editorial Policies and the Editorial Policy Checklist.

Statistics

For all statistical analyses, confirm that the following items are present in the figure legend, table legend, main text, or Methods section.

n/a	Confirmed	
☐	☒	The exact sample size (n) for each experimental group/condition, given as a discrete number and unit of measurement
☐	☒	A statement on whether measurements were taken from distinct samples or whether the same sample was measured repeatedly
☐	☒	The statistical test(s) used AND whether they are one- or two-sided<br>*Only common tests should be described solely by name; describe more complex techniques in the Methods section.*
☐	☒	A description of all covariates tested
☐	☒	A description of any assumptions or corrections, such as tests of normality and adjustment for multiple comparisons
☐	☒	A full description of the statistical parameters including central tendency (e.g. means) or other basic estimates (e.g. regression coefficient) AND variation (e.g. standard deviation) or associated estimates of uncertainty (e.g. confidence intervals)
☐	☒	For null hypothesis testing, the test statistic (e.g. F, t, r) with confidence intervals, effect sizes, degrees of freedom and P value noted<br>*Give P values as exact values whenever suitable.*
☒	☐	For Bayesian analysis, information on the choice of priors and Markov chain Monte Carlo settings
☒	☐	For hierarchical and complex designs, identification of the appropriate level for tests and full reporting of outcomes
☒	☐	Estimates of effect sizes (e.g. Cohen's d, Pearson's r), indicating how they were calculated

Our web collection on statistics for biologists contains articles on many of the points above.

Software and code

Policy information about availability of computer code

Data collection	Microsoft® Excel® for Microsoft 365 MSO (Version 2311 Build 16.0.17029.20028) 64 Bit
Data analysis	GraphPad Prism 10.1.0 (316)

For manuscripts utilizing custom algorithms or software that are central to the research but not yet described in published literature, software must be made available to editors and reviewers. We strongly encourage code deposition in a community repository (e.g. GitHub). See the Nature Portfolio guidelines for submitting code & software for further information.

Data

Policy information about availability of data

All manuscripts must include a data availability statement. This statement should provide the following information, where applicable:

- Accession codes, unique identifiers, or web links for publicly available datasets
- A description of any restrictions on data availability
- For clinical datasets or third party data, please ensure that the statement adheres to our policy

All data is provided within this manuscript and the corresponding source data files. Previously generated sequencing data (for virus isolates) is referenced as accession code in the materials and methods section.

Research involving human participants, their data, or biological material

Policy information about studies with human participants or human data. See also policy information about sex, gender (identity/presentation), and sexual orientation and race, ethnicity and racism.

Reporting on sex and gender	N/A
Reporting on race, ethnicity, or other socially relevant groupings	N/A
Population characteristics	N/A
Recruitment	N/A
Ethics oversight	N/A

Note that full information on the approval of the study protocol must also be provided in the manuscript.

Field-specific reporting

Please select the one below that is the best fit for your research. If you are not sure, read the appropriate sections before making your selection.

☒ Life sciences ☐ Behavioural & social sciences ☐ Ecological, evolutionary & environmental sciences

For a reference copy of the document with all sections, see nature.com/documents/nr-reporting-summary-flat.pdf

Life sciences study design

All studies must disclose on these points even when the disclosure is negative.

Sample size	No statistical method was used to predetermine sample size. Instead, we selected a sample size of n = 3-6 animals per time point and treatment based on our previous experience with SARS-CoV-2 vaccination and infection of Syrian hamsters. To adhere to the 3R principle, we reduced the number of animals used in this study to the minimum that had been experimentally determined in our previous studies (DOI: 10.1016/j.celrep.2021.109493, DOI: 10.1126/sciadv.abk0172, DOI: 10.1016/j.ymthe.2023.05.004 and DOI: 10.1038/s41564-023-01352-8).
Data exclusions	No data was excluded.
Replication	We performed two independend animal experiments for the purpose of this study, one to study transmission of ancestral variant B.1, the second to study transmission of BA.5. With male 54 hamsters in each study arm, the total amount of animals used for the purpose of this study is 108 male hamsters. The results of these two independently performed studies are in good agreement and substantionate our findings. All virological assays (titrations, qPCR, SNT and ELISA) were performed in duplicates (technical replicates) for each animal included in the study. All replication attempts were successful. The respective mean values were then used as individual data points for biological replicates (individual animals). To ensure robust data analysis, we reported data generated from a minimum of 3 biological replicates (individual animals) for all analyses.
Randomization	Hamsters were randomly assigned to groups for all experiments. In other experiments, sample allocation was not random. Experiments were controlled with appropriate measures, such as standard curves and known positive and negative controls.
Blinding	All animal experimentation was performed blindly. To this end, animals were assigned consecutive numbers, all personal conducting the experiments and subsequent analysis, including histopathology, virological examination and serology were only aware of the respective animal number, not of the treatment the animal had received. The person performing vaccinations was aware of the vaccine each hamster received, which is technically necessary due to different handling and application routes. However, this person was different from the personal that monitored and weighed the animals or performed further analyses.

Reporting for specific materials, systems and methods

We require information from authors about some types of materials, experimental systems and methods used in many studies. Here, indicate whether each material, system or method listed is relevant to your study. If you are not sure if a list item applies to your research, read the appropriate section before selecting a response.

Materials & experimental systems

n/a	Involved in the study	
☐	☒	Antibodies
☐	☒	Eukaryotic cell lines
☒	☐	Palaeontology and archaeology
☐	☒	Animals and other organisms
☒	☐	Clinical data
☒	☐	Dual use research of concern
☒	☐	Plants

Methods

n/a	Involved in the study	
☒	☐	ChIP-seq
☒	☐	Flow cytometry
☒	☐	MRI-based neuroimaging

Antibodies

Antibodies used	ELISA: Rabbit Anti-Hamster lgA, HRP-conjugated (Brookwood Biomedical, Jemison, AL) sab3003a, LOT: 004/005, used at 1:750 dilution EILSA: Goat anti-Syrian Hamster lgG (H+L), HRP-conjugated, (lnvitrogen, Fisher Scientific, Schwerte, Germany) PA129626, LOT: AB_10985385 used at 1: 1000 dilution.<br>IHC: Anti-SARS-CoV-1/2 NP mouse monoclonal antibody, Clone #05, (Sino Biological lnc.; Beijing, China) 40143-MM05, LOT: NP_828858.1, used at 1:500 dilution.<br>IHC: Biotinylated Goat-Anti-Mouse lgG antibody (Vector Laboratories, Burlingame, California USA) BP-9200-50, LOT: ZB9202, used at 1:200 dilution.<br>Immunofluorescence staining: Primary polyclonal anti-SARS Coronaviurs NP antibody (lnvitrogen, Fisher Scientific, Schwerte, Germany) PA1-41098, LOT: Yi3782596/Yl3787824, used at 1:2500. Goat anti-Rabbit IgG-AlexaFluor 488 (Invitrogen, Fisher Scientific, Schwerte, Germany) PA5-16891, LOT: 2557379, used at 1:2500.
Validation	ELISA: Brookwood Biomedical, sab3003a was validated on unrelated hamster serum and nasal wash samples in our lab. lnvitrogen PA129626 was validated by the manufacturer: https://www.thermofisher.com/antibody/product/Goat-anti-Syrian-Hamster-lgG-H-L-Secondary-Antibody-Polyclonal/PAl -2962 6 IHC: Anti-SARS-CoV-1/2 NP mouse monoclonal antibody, Sino Biological 40143-MM05, validated by the manufacturer used for SARS-CoV-2 IHC by others (DOI: https://doi.org/10.1038/s41467-021-22580-8). The secondary antibody Vector Laboratories BP-9200-50 has been used for detection of mouse lgG by IHC in multiple previous publications (see: https://vectorlabs.com/immpress-hrp-anti-mouse-igg-rat-adsorbed-peroxidase-polymer-detection-kit.html).

Eukaryotic cell lines

Policy information about cell lines and Sex and Gender in Research

Cell line source(s)	Vero E6 ATCC (ATCC CRL-1586)<br>Vero E6 TMPRSS (NIBSC 100978)<br>Calu-3 (ATCC HTB-55)
Authentication	Vero E6 cells and CaLu-3 cells were authentificated by Deutsche Sammlung von Mikroorganismen und Zellkulturen after experiments were finished. Authentification confirmed the identity of the cells. No authentification was performed for other cells lines. Cells were used exclusively for SARS-CoV-2 preparation and/or plaque assays.
Mycoplasma contamination	All cells were tested negative for mycoplasma by PCR prior to start of experiments.
Commonly misidentified lines<br>(See ICLAC register)	None.

Animals and other research organisms

Policy information about studies involving animals; ARRIVE guidelines recommended for reporting animal research, and Sex and Gender in Research

Laboratory animals	A total of 108 Mesocricetus auratus (Syrian hamster, also known as golden hamster), Janvier labs, Outbred hamster, RjHan:AURA, 5 to 10 weeks of age at experiment start.
Wild animals	No wild animals were used in this study.
Reporting on sex	To enable co-housing, transmission experiment were performed using male animals only.
Field-collected samples	No field-collected samples were used in this study.
Ethics oversight	All animal experiments were performed in compliance with relevant institutional, national, and international guidelines for care and humane use of animal and approved by the Landesamt für Gesundheit und Soziales in Berlin, Germany (permit number 0086/20).

Note that full information on the approval of the study protocol must also be provided in the manuscript.
